# Supplementary material for: Novel Driver Strength Index highlights important cancer genes in TCGA PanCanAtlas patients
Source: PeerJ. 2022 Aug 11;10:e13860. doi: 10.7717/peerj.13860 (PMC9375969; doi:10.7717/peerj.13860)
Supplement: Supplemental Information 2 [file peerj-10-13860-s002.zip › COHORTS/genes plots/stage/2021_8_16_14_9_distribution_stage_males_III.pdf]

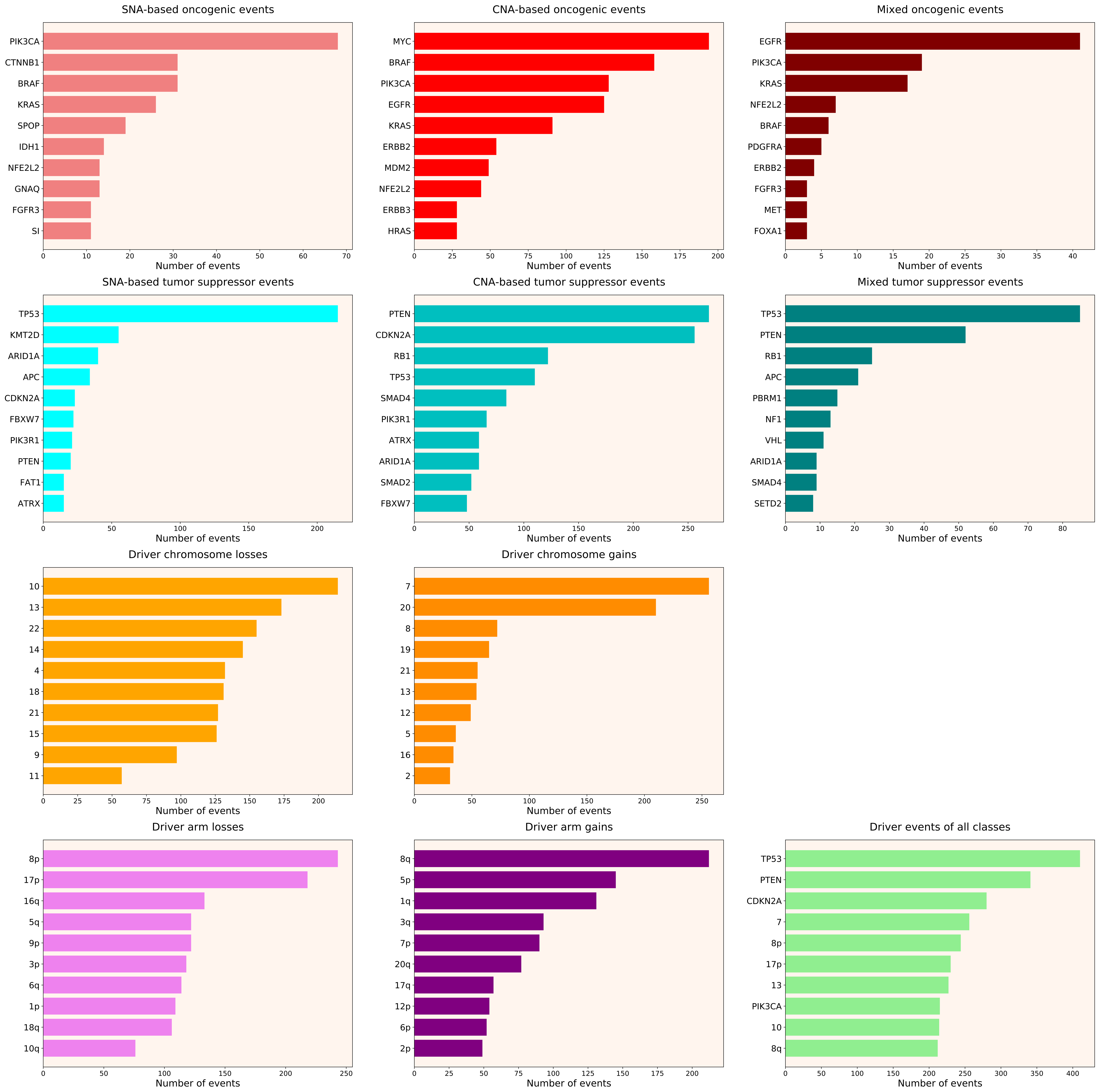

SNA-based oncogenic events
PIK3CA
CTNNB1
BRAF
KRAS
SPOP
IDH1
NFE2L2
GNAQ
FGFR3
SI
0 10 20 30 40 50 60 70
Number of events
CNA-based oncogenic events
MYC
BRAF
PIK3CA
EGFR
KRAS
ERBB2
MDM2
NFE2L2
ERBB3
HRAS
0 25 50 75 100 125 150 175 200
Number of events
Mixed oncogenic events
EGFR
PIK3CA
KRAS
NFE2L2
BRAF
PDGFRA
ERBB2
FGFR3
MET
FOXA1
0 5 10 15 20 25 30 35 40
Number of events
SNA-based tumor suppressor events
TP53
KMT2D
ARID1A
APC
CDKN2A
FBXW7
PIK3R1
PTEN
FAT1
ATRX
0 50 100 150 200
Number of events
CNA-based tumor suppressor events
PTEN
CDKN2A
RB1
TP53
SMAD4
PIK3R1
ATRX
ARID1A
SMAD2
FBXW7
0 50 100 150 200 250
Number of events
Mixed tumor suppressor events
TP53
PTEN
RB1
APC
PBRM1
NF1
VHL
ARID1A
SMAD4
SETD2
0 10 20 30 40 50 60 70 80
Number of events
Driver chromosome losses
10
13
22
14
4
18
21
15
9
11
0 25 50 75 100 125 150 175 200
Number of events
Driver chromosome gains
7
20
8
19
21
13
12
5
16
2
0 50 100 150 200 250
Number of events
Driver arm losses
8p
17p
16q
5q
9p
3p
6q
1p
18q
10q
0 50 100 150 200 250
Number of events
Driver arm gains
8q
5p
1q
3q
7p
20q
17q
12p
6p
2p
0 25 50 75 100 125 150 175 200
Number of events
Driver events of all classes
TP53
PTEN
CDKN2A
7
8p
17p
13
PIK3CA
10
8q
0 50 100 150 200 250 300 350 400
Number of events
